# 2290. Neurological Symptoms of Long - COVID in Mexican Ambulatory Adults with Mild COVID-19

**DOI:** 10.1093/ofid/ofad500.1912

**Published:** 2023-11-27

**Authors:** Jorge-Baruch Díaz-Ramírez, Jorge Bryan Ortega Marquez, Yazmín González-Valadez, Luis-Alberto Cortazar-Maldonado, José-Antonio Morales-Fernández, Miguel A Pérez-Sastré, Roberto Martínez Rojas, Leopoldo D Trujillo García, Martha Fragoso García, Diana Mondragón Salinas, Lissete Navarrete Hernández, Diana Pérez Acosta, Miguel Leonardo García-León, Patricia Bautista-Carbajal, Antonio H Ángel-Ambrocio, Jorge Camacho-Morales, Antonio R Villa, Rosa-María Wong-Chew

**Affiliations:** Universidad Nacional Autónoma de México, Mexico City, Distrito Federal, Mexico; National Autonomous University of Mexico, Coyoacan, Distrito Federal, Mexico; Universidad Nacional Autónoma de México, Mexico City, Distrito Federal, Mexico; Universidad Nacional Autónoma de México, Mexico City, Distrito Federal, Mexico; Universidad Nacional Autónoma de México, Mexico City, Distrito Federal, Mexico; Universidad Nacional Autonoma de México, Mexico City, Distrito Federal, Mexico; Universidad Nacional Autónoma de México, Mexico City, Distrito Federal, Mexico; Universidad Nacional Autónoma de México, Mexico City, Distrito Federal, Mexico; Universidad Nacional Autónoma de México, Mexico City, Distrito Federal, Mexico; Universidad Nacional Autónoma De México, Mexico City, Distrito Federal, Mexico; Universidad Nacional Autónoma de México, Mexico City, Distrito Federal, Mexico; Universidad Nacional Autónoma de México, Mexico City, Distrito Federal, Mexico; Universidad Nacional Autonoma de Mexico, Mexico City, Distrito Federal, Mexico; Universidad Nacional Autónoma de México, Mexico City, Distrito Federal, Mexico; Universidad Nacional Autonoma de Mexico, Mexico City, Distrito Federal, Mexico; Departamento de Informática Biomédica, Facultad de Medicina, UNAM, CDMX, Distrito Federal, Mexico; Universidad Nacional Autónoma de México, School of Medicine, Mexico, Distrito Federal, Mexico; Facultad de Medicina, Universidad Nacional Autónoma de México, Mexico, Distrito Federal, Mexico

## Abstract

**Background:**

The COVID-19 pandemic represents one of the greatest public health crises in this century . Since the beginning of the pandemic there were reports of persistence of symptoms after an average recovery period of 2-5 weeks. Long-COVID syndrome was defined as a continuation or onset of symptoms within 3 months of the initial infection, lasting for at least 2 months with no explanation. There are few studies focused on neurological symptoms of long COVID in ambulatory patients who suffered a mild disease.

**Methods:**

We conducted a prospective cohort study with a univariate and Kaplan-Meier survival analysis with data obtained between January 2020 to November 2022 from ambulatory adults with mild to moderate COVID-19 diagnosed at our UNAM Faculty of Medicine COVID-19 Diagnostic Center in Mexico City. Patients were systematically called every 15 days during 6 months to evaluate self-perceived neurological symptoms after being diagnosed.

**Figure:**
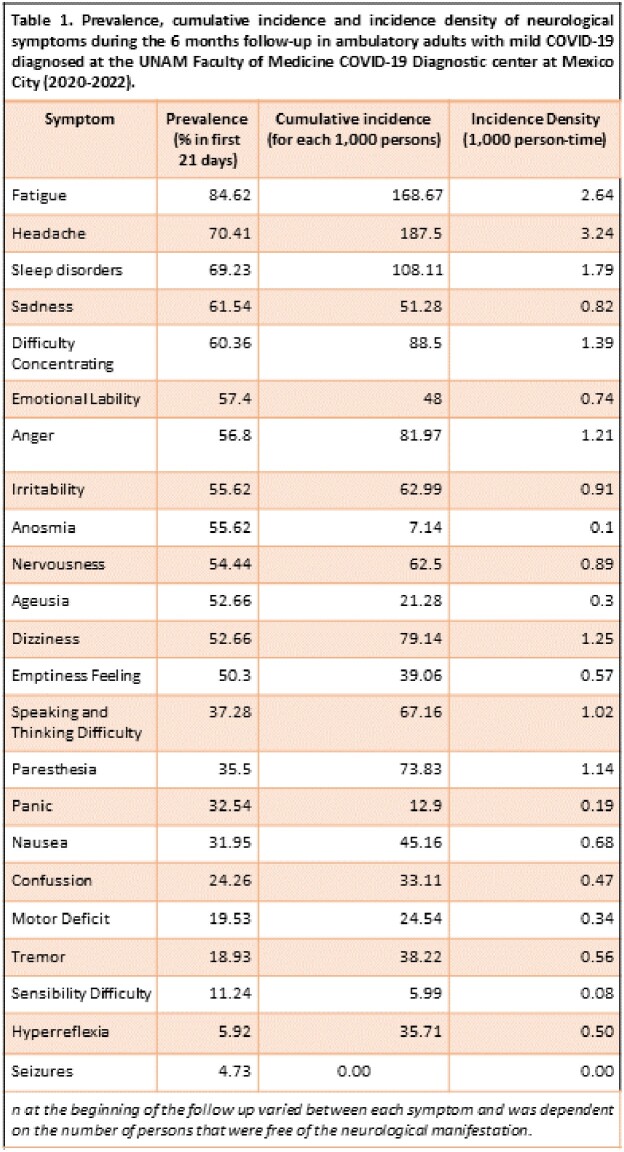

**Figure:**
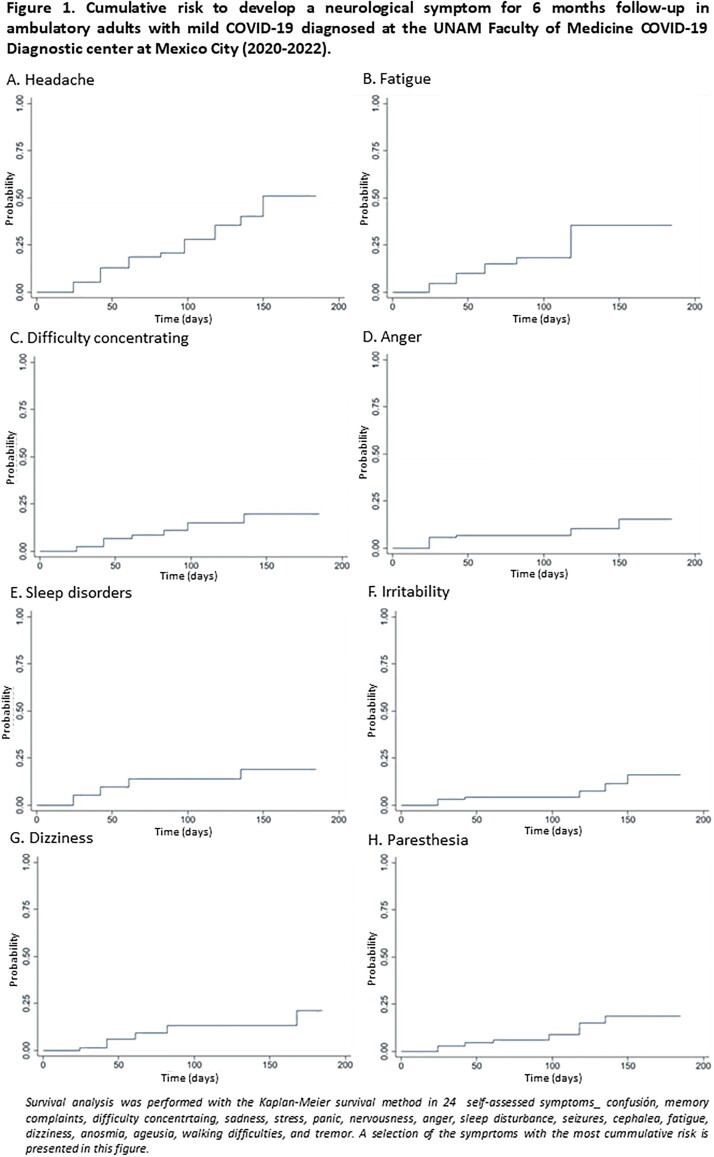

**Results:**

We assessed 169 COVID 19 patients, the mean age was 39 (SD 15) years, 42% were men, 97% were Mexican, 90% developed a new neurologic symptom after the COVID-19 acute period. The most common symptoms reported during the follow up were in the first 21 days: fatigue (84%), headache (70%), sleep disorders (69%), sadness (61%), concentration difficulties (60%), emotional liability (57%), anger (56%) and irritability (55%) among others. The highest cumulative incidence (per 1,000 persons) was for headache (187), fatigue (168), sleep disorders (108), concentration difficulties (88), anger (81), dizziness (79), paresthesia (73), speaking and thinking difficulties (67) among others.

The survival analysis showed an increased cumulative probability to develop neurological symptoms during the 6 months follow-up, with the highest risk for headache (50%) and fatigue (40%).

**Conclusion:**

The probability of presenting neurologic symptoms after suffering a mild COVID 19 disease increased during the 6 months follow up. Additionally, most symptoms were of new onset after the initial recovery from an acute mild COVID-19 episode or persisted from the initial illness and fluctuated over the follow-up, except for seizures that were present as an isolated symptom during acute phase and absent during the follow-up.

**Disclosures:**

**All Authors**: No reported disclosures

